# British Association

**Published:** 1840-10-10

**Authors:** 


### MEDICAL EXAMINER.

DEVOTED TO MEDICINE, SURGERY, AND THE COLLATERAL SCIENCES.

No. 41.] PHILADELPHIA, SATURDAY, OCTOBER 10, 1840. [Vol. III.

### FOREIGN CORRESPONDENCE.

*British Association.—Report on the Motions and Sounds of the Heart.—Chemical Report.*

To the Editors of the Medical Examiner.

The tenth meeting of the British Association has been just concluded at Glasgow. In the Medical Section little of interest was done. The London Committee "on the Motions and Sounds of the Heart," appointed some years since to investigate this intricate and important subject, presented their Report. This was so voluminous a paper, that the conclusions, separately drawn up, were alone submitted to the Section. The premises on which these were based, were of course unknown, and will be made public through the transactions of the Association, in a form inaccessible to nine-tenths of even our insular confrères, and at some future period when the results themselves have long been forgotten. The object, moreover, of the labours of the present Committee was, if we mistake not, to repeat the series of experiments made by the Dublin Committee, with the addition of such others as might be thought proper, in order to test the accuracy of the results obtained by the latter. Five years have elapsed since the report of the Dublin committee, and the difficulty of comparison has become great, if not almost impracticable.

We shall now present our readers with the seventeen conclusions of the London committee, hoping, before long, to be enabled to offer them some of the details, and, until then, abstaining from either endorsing or denying these Anglican articles of faith.

1stly. That the order of the motions of the auricles and ventricles, is by continuous succession rather than by alternation.

2dly. That the visible systolic, and diastolic, are first perceived at the bases or fixed parts of the cavities, viz., in the auricles at the sinuses, and in the ventricles at the *fundus cordis*, and that the free parts are brought into full action after the other parts.

3dly. That in systole the heart is diminished in all directions and that its long axis is invariable shortened.

4thly. That the normal systole of the auricles is energetic and almost instantaneous, and quite universal.

5thly. That the systole of the ventricles is gradual in its development, and complex in its phenomena, attributable to the contraction of the muscular parietes, and to resistance on the part of the fluids.

6thly. That the pulsation of the veins is the same, at least in some animals (!)—both active and passive, and that the latter is attributable to reflux from the auricles during their systole.

*Motions.*—7thly. That the normal pulsation or throb, appears to be caused mainly by the undulatory and eccentric resistance to muscular compression, exerted by the blood in systole, and to be in no degree attributable to any blow, stroke, or other form of impulse implying change of place, or any other change than that of shape, and of parietal thickness and tension in the heart. This cardiac impulse is evident in the exposed heart.

8thly. That the arterial diastole, or pulse, almost every where outside the pericardium, perceptibly succeeds to the cardiac systole, though near the heart the interval is very brief.

*Sounds.*—9thly. That the first sound depends partly, but in a slight degree, on the abrupt closure and transitory tension of the auriculo-ventricular valves, which give to this sound its sharp, well-defined beginning; but that the first sound is mainly attributable to cardio-muscular tension alone, and that its prolonged duration is probably owing to the progressive character of the normal systolic effort from fundus to apex, and that this sound is probably in no degree or condition attributable to any blow or stroke against the ribs.

10thly. That the auricular systole is attended by an intrinsic sound resembling that of the ventricles, but more short, obtuse and feeble.

11thly. That the sounds of friction in pericarditis may, when well marked, and under ordinary circumstances, be expected to be double at least, and that they may be triple, or more. In its systolé, each cavity of the heart moves so as to cause a friction in one direction of its attached laminæ, against the adjacent free laminæ of the pericardium, and in its dyastole a pericardial friction is caused by each cavity in an opposite direction; and as the auricles move to and fro, independently of the ventricles, the normal pericardial frictions must be quadruple, or double with the auricles, and double with the ventricles. If, therefore, these frictions be rendered sonorous by the interposition of any rough snbstance between the rubbing surfaces, as lymph, for example, and, supposing the heart's action sufficiently vigorous, we might anticipate a duplication of murmurs at least—the one systolic, the other diastolic. And this must be the principal element in the acoustic diagnosis.

12thly. That the normal sounds of the heart are much liable to modification, by deviations from the normal standard in the order, force, and excitability of the carneæ columnæ, and the other contracting parts, governing and influencing the action of the valves, and the closure or opening again of the orifices of the ventricles; and this dependence on conditions, excluding structural defect, is so considerable that the second may be for a time variously modified or marked by strange murmurs, or even apparently suppressed, in consequence of changes in the solids of purely a dynamic character, or caused by humoral defect in consequence of hæmorrhage from the introduction of poison into the veins. And the first cardiac sound, though never wholly wanting during the active existence of the heart, may still, under similar circumstances, present various abnormal features.

13thly. That the peculiar sounds occurring in pericarditis, and attributable to pericardial frictions, are not referable only to vascular turgescence, or dryness of the pericardium, but to lymph effused by, and adhering to that membrane, or other equivalent obstacle, to the easy and noiseless gliding of the two pericardial laminæ.

14thly. That the ventricles are of equal capacity during life, and that the inequality observable after death is an illusion explained long since by Harvey.

15thly. That the suction influence on the venous circulation, attributed by writers to respiration, is well founded.

16thly. That the action of the long muscles, and more especially those of the abdominal parietes, is attended with an intrinsic sound.

17thly. That the sounds of the heart, as well as the motions, are governed by the same law in all warm-blooded animals hitherto examined. That their causation follows, likewise, the same law as those of man—the first sound being mainly muscular, and the second, probably, exclusively valvular. Also, that there is the same causation and mutual relation of the cardiac and arterial pulsations.

Sir Charles Bell remarked, that it was not a little singular, that no reference was made to the opinion of so distinguished a physiologist as Hunter, as to the efficient cause of the cardiac impulse, when the apex of the heart strikes on the parietes of the chest,—the effort made by the arch of the aorta to elongate itself upon receiving the column of blood, forced into it by the ventricular systole, by which effort the axis of the heart's direction is changed, the apex being forced up against the chest. In accounting for the sounds, there was no mention made of the rush of blood through those irregular masses the columnæ carneæ, and their tendinous connections with the valvular apparatus.

In the Chemical Section, Prof. Schafhaenth read a paper on the discovery of a "new compound of Arsenious and Sulphuric Acids," obtained from the escaping smoke of copper calcining furnaces near Swansea, in South Wales. Here, too, was another instance of an anhydrous crystallized body deposited under the presence of water only, affording a remarkable proof of the unlimited number of different forms of combination, which might be produced even in inorganic nature, by bringing chemical substances in contact under varying circumstances. The copper ores smelted in South Wales are, for the most part, copper pyrites, mixed with iron pyrites; gray copper ore—a mixture in which we have the sulphurets of copper, iron, arsenic, antimony, cobalt, nickel, zinc, and tin, invariably found together. During the calcining process, the sulphur and arsenic escape in the form of sulphurous and arsenious acid. These destroy all vegetation around the furnaces for miles, whereas they are perfectly innocuous to animal life. By bringing the escaping fumes in contact with steam, and forcing it through burning charcoal, or by subjecting it to great pressure, the new solid compound is deposited on the cool surfaces of the chambers connected with the calcining furnace, in beautiful crystallized leaves or tables, belonging, perhaps, to the same class as Wöhler's dimorphic modification of the crystallization of arsenious acid, the regular of which belongs to the octahedron. It consists in 100 parts of

| | |
|---|---|
| 68.250 | Arsenious Acid, |
| 27.643 | Sulphuric Acid, |
| 3.029 | Protoxide of Iron, |
| 0.420 | Oxide of Copper, |
| 0.656 | Oxide of Nickel. |
| 99.998 | |

Corresponding to

| | |
|---|---|
| 51.741 | Metallic Arsenic, |
| 11.095 | Sulphur, |
| 2.339 | Iron, |
| 0.336 | Copper, |
| 0.516 | Nickel, |
| 33.971 | Oxygen. |
| 99.998 | |

These crystals attracted moisture from the air, with great rapidity and with evolution of heat, corroding animal and vegetable substances as powerfully as concentrated sulphuric acid. Their taste was pure, but very sour, resembling sulphuric acid, and dissolved in water, the remainder of 100 parts of these crystals was 17.436 grains only. The crystals remained perfectly retained, only their appearance was changed from transparent into opaque. Their chemical composition was found to be

| | |
|---|---|
| 16.778 | grains of Arsenious acid, |
| 0.656 | Oxide of Nickel. |
| 17.434. | |

What the water had dissolved, consisted of

| | |
|---|---|
| 51.472 | Arsenious Acid, |
| 27.643 | Sulphuric Acid, |
| 3.029 | Protoxide of Iron, |
| 0.420 | Oxide of Copper. |
| 82.564 | |

A remarkable change occurred during the formation of this compound—the conversion of sulphurous into sulphuric acid, as well as the presence of iron, copper, and nickel in a deposit from gaseous matter. This is the only definite compound of arsenic acid with another acid known, except those with the two organic acids—tartaric and paratartaric.

Prof. Gregory, in a communication "on the pre-existence of Urea in Uric Acid," stated that Liebig and Wöhler having, by the action of peroxide of lead on uric acid, obtained from it oxalic acid, allantoine, and urea, and that they considered urea as existing in the uric acid in combination with urile, he (Prof. Gregory) having found urea, unlike most organic substances, resist the oxidizing agency of permanganate of potash, imagined, that if urea could be obtained from uric acid by the action of that salt, it would strengthen, materially, the opinion in favor of its pre-existence. For if only the elements of urea were present, the oxidizing agency of the permanganate would most likely prevent its formation. On the experiment being tried, a large quantity of urea was obtained along with oxalic acid, and a new acid; the latter, probably, was formed by the oxidization of allantoine. Prof. Gregory, in the course of the paper, described the acetate of urea, a salt formed in the course of his experiments.

London, September 26, 1840.

